# The Enforcement of Intimate Image Offences and the Effectiveness of Victim Services in Taiwan: A Qualitative Study Using Reflexive Thematic Analysis

**DOI:** 10.3390/ijerph23040525

**Published:** 2026-04-18

**Authors:** Wen-Ling Hung

**Affiliations:** Department of Criminal Justice, Ming Chuan University, Taoyuan 333, Taiwan; wenling@mail.mcu.edu.tw

**Keywords:** online gender-based violence, intimate image offences, technology-facilitated sexual violence, victim support services, reflexive thematic analysis, Taiwan, criminal justice, trauma-informed practice

## Abstract

**Highlights:**

**Public health relevance—How does this work relate to a public health issue?**

- Intimate image offences constitute a form of technology-facilitated gender-based violence that is associated with trauma, anxiety, depression, and broader psychosocial harm.
- This study examines how Taiwan’s recent legal reforms on digital sexual violence are being implemented in frontline practice and how these responses affect victim protection.

**Public health significance—Why is this work of significance to public health?**

- Based on qualitative interviews with 20 frontline professionals, this study identifies multi-level barriers—public awareness deficits, legal fragmentation, and procedural challenges—that limit effective enforcement and victim support.
- The findings show that legal reform alone is insufficient; effective responses depend on the alignment of social norms, institutional capacity, and trauma-informed victim-centred practice.

**Public health implications—What are the key implications or messages for practitioners, policy makers and/or researchers in public health?**

- Public education, digital literacy promotion, and anti-victim-blaming initiatives are needed to strengthen prevention, reporting, and early intervention.
- Policymakers and practitioners should prioritise rapid takedown and digital evidence-preservation mechanisms, stronger inter-agency coordination, and integrated psychosocial and legal support for victims.

**Abstract:**

(1) Background: The non-consensual dissemination of intimate images constitutes a severe form of online gender-based violence (OGBV) that inflicts profound harm on victims’ sexual privacy, psychological well-being, and social functioning. Taiwan enacted comprehensive legislative reforms in 2023—commonly referred to as the “Four Acts on Sexual Violence Prevention”—to strengthen criminal responses and expand victim protection mechanisms. However, the extent to which these reforms have translated into effective frontline practice remains insufficiently examined. (2) Methods: This qualitative study employed reflexive thematic analysis to investigate frontline professionals’ experiences with enforcing intimate image offence legislation and delivering victim support services. Semi-structured, in-depth interviews were conducted with 20 practitioners, including social workers, police officers, prosecutors, and lawyers. (3) Results: Three superordinate themes emerged across macro, meso, and micro structural levels. At the macro level, limited public awareness and persistent victim-blaming attitudes undermine prevention, help-seeking, and reporting. At the meso level, legislative fragmentation, challenges in preserving and analysing digital evidence, and inter-agency coordination gaps constrain enforcement capacity. At the micro level, procedural delays, risks of secondary victimization, and perceived inadequacies in compensation and support mechanisms weaken victims’ trust in institutional responses. (4) Conclusions: While Taiwan’s legislative reforms represent a significant institutional advancement, legal reform alone is insufficient to address digital sexual violence effectively. Comprehensive responses require integrated public education initiatives, enhanced inter-agency coordination, strengthened digital investigation capacity, and trauma-informed victim protection practices across all structural levels. In particular, the findings underscore an urgent public health need to establish rapid digital evidence preservation and takedown mechanisms to limit the proliferation of non-consensual sexual images and mitigate the associated mental health harms among victims.

## 1. Introduction

The rapid proliferation of digital technologies has been accompanied by an increase in online gender-based violence (OGBV). Among its various forms, the non-consensual dissemination of intimate images has been widely documented as a serious infringement of personal privacy and has been associated with adverse psychological outcomes, including anxiety, depression, and trauma-related symptoms [1,2]. As smartphones and social media platforms become increasingly integrated into everyday life, the forms and reach of such abuse continue to evolve, creating complex challenges for legal systems, public health responses, and social support services across different jurisdictions [3].

OGBV is technology-facilitated abuse (TFA), which refers to a range of harmful behaviors carried out through digital means, such as the unauthorized sharing of intimate content, online harassment, cyberstalking, and sexual extortion [1]. The instantaneous and transnational characteristics of digital communication have been shown to intensify victims’ exposure to harm and to complicate recovery processes [4]. At the institutional level, these characteristics are also associated with difficulties in coordinating responses across regulatory, service, and support systems, particularly in contexts characterized by regulatory complexity, fragmented jurisdictional authority, and the dispersion of abusive content across multiple online platforms [5].

In response to the growing recognition of digital sexual violence, Taiwan implemented a set of legislative reforms in 2023 to address emerging forms of digital gender-based violence, including the non-consensual dissemination of intimate images [6,7]. Commonly referred to as the “Four Acts on Sexual Violence Prevention,” these amendments were finalized in February 2023 and came into full effect in July of the same year, encompassing revisions to the Criminal Code, the Sexual Assault Crime Prevention Act, the Child and Youth Sexual Exploitation Prevention Act, and the Crime Victim Rights Protection Act [7]. The key elements of these reforms include the introduction of a legal definition for “intimate images,” the establishment of criminal provisions related to offenses against sexual privacy, the strengthening of criminal liability for non-consensual dissemination, the clarification of mandatory takedown mechanisms for intimate images, and the expansion of judicial remedies and protective measures for victims—collectively aimed at enhancing the comprehensiveness of institutional responses [7].

While these reforms represent a significant institutional development, existing observations suggest that their implementation in practice may be uneven. Challenges related to procedural complexity, fragmented legal provisions, and cross-agency coordination have been noted as factors that may influence how frontline professionals deliver support and manage cases involving intimate image offences [3]. This gap underscores the need for further examination of the relationship between institutional design and practical implementation.

Despite increasing scholarly attention to intimate image abuse, much of the empirical literature on institutional and service responses has focused on Western contexts, with comparatively limited examination of East Asian jurisdictions, including Taiwan [7]. In addition, research integrating victimological perspectives with digital violence studies and public health frameworks—particularly those centered on the experiences of frontline practitioners—remains relatively limited.

The present study addresses these gaps by examining the implementation of Taiwan’s recent legislative reforms and exploring the effectiveness of victim support services from the perspectives of professionals directly engaged in prevention and enforcement. Employing qualitative interviews with social workers, legal professionals, prosecutors, and police officers, this study aims to provide contextually grounded insights into the persistent barriers, institutional dynamics, and potential opportunities for reform that shape responses to OGBV in a rapidly evolving digital environment.

In 2023, Taiwan amended four major statutes on sexual violence to address digital sexual and gender-based violence, including non-consensual dissemination of sexual images, deepfake sexual imagery, and online exploitation of minors. Table 1 summarizes the core focus and key 2023 amendments of these “Four Acts on Sexual Violence Prevention” for international readers.

From a public health perspective, technology-facilitated sexual violence is associated with substantial trauma, anxiety, depression, and longer-term psychosocial distress, underscoring the need to examine institutional responses as part of broader prevention and mental health systems.

Accordingly, this study adopts a public health lens to explore how legal and institutional responses to intimate image abuse may mitigate or exacerbate mental health harms and shape access to prevention and support.

## 2. Literature Review

The literature review for this study is organized into three thematic areas: (1) theories related to victimology, (2) digital technology and gender-based violence, and (3) psychological impacts and victim fear. This structure is designed to integrate core theoretical concepts, summarize recent international research trends, and provide the theoretical foundation for the study’s research motivation and analytical framework [3].

### 2.1. Theories Related to Victimology

Victimology provides an important analytical framework for understanding both the formation of risk and institutional responses in cases of OGBV. In this context, OGBV refers to harmful acts perpetrated through digital technologies, including the non-consensual dissemination of intimate images [2]. From this perspective, victimological theories help to explain how structural conditions, routine activities, and power relations shape patterns of victimization and inform the design of prevention and intervention strategies [8].

#### 2.1.1. Routine Activity Theory (RAT)

Routine Activity Theory (RAT) posits that victimization is likely to occur when three elements converge in the same time and space: a motivated offender, a suitable target, and the absence of capable guardianship [8]. In digital environments, “routine activities” encompass everyday online behaviors such as social media use, instant messaging, image sharing, and participation in online communities. These activities not only shape individuals’ exposure to potential offenders but also influence the availability, replicability, and visibility of intimate images as potential targets [3,9].

In the context of intimate image offences, RAT highlights how platform design and norms of digital interaction may inadvertently increase target suitability and weaken institutional and social guardianship. Features such as the high persistence of digital data, the low cost of copying and forwarding images, insufficient default privacy settings, and limited identity verification and accountability mechanisms reduce the effort required to disseminate images without consent and enable rapid expansion of audiences once images begin to circulate [3,10]. At the same time, victimological scholarship repeatedly emphasizes that such analyses should not be used to blame individuals for their victimization. Compared with any single victim’s behavior, factors such as platform governance, legal protections, school and family support systems, and broader gendered power structures constitute more direct and critical determinants of risk and protection [3,11].

Recent extensions of victimological theory have also begun to address the dynamic overlap between victim and offender roles in image-based sexual abuse. Empirical studies on the non-consensual dissemination of sexual images (NCDSI) indicate that prior experiences of online victimization, engagement in high-risk online routine activities, and exposure to unsolicited sexual images are associated both with subsequent victimization and with perpetration, suggesting that these phenomena cannot be understood through a simple dichotomy between victims and offenders, but rather involve a potential victim–offender overlap [12,13].

Additionally, victimology provides an important lens for understanding secondary victimization, risk perception, and help-seeking in intimate image cases [14,15]. Research on fear of cybercrime shows that prior online victimization, perceived vulnerability, and broader social and environmental factors—such as low collective efficacy and disorder in online spaces—significantly shape individuals’ risk assessments and levels of fear [16,17]. In the context of intimate image misuse, victims often anticipate disbelief, blame, or minimization of the incident when approaching authorities, which suppresses disclosure and contributes to persistent underreporting [14,15].

These patterns align with victimological critiques of the uncritical use of “victim precipitation” frameworks in sexual violence research, where excessive emphasis on victims’ behavior can reinforce stigma and weaken institutional accountability [18,19]. From a public policy and practice perspective, these findings underscore the importance of establishing trauma-informed and non-blaming response procedures [20], clearly recognizing image-based sexual abuse in law [21], and strengthening specialized training for frontline professionals so that they can function as capable guardians rather than sources of secondary harm [22,23].

#### 2.1.2. Lifestyle–Exposure Theory and Empirical Evidence from Taiwan

Lifestyle–Exposure Theory extends the perspective of Routine Activity Theory by further emphasizing how individuals’ patterns of daily activities shape their differential exposure to motivated offenders and high-risk environments [24]. In the context of OGBV, this theory is particularly useful for understanding how specific online lifestyles—such as intensive social media use, frequent image sharing, and participation in dating or instant messaging applications—structure opportunities for technology-facilitated abuse [25]. Related research indicates that online routine activities do not occur randomly; rather, they are shaped by age, gender, social roles, and broader socio-cultural norms, which in turn influence both risk and protection in cyberspace [26,27].

Although direct empirical applications of Lifestyle–Exposure Theory to OGBV research in Taiwan remain relatively limited, recent studies on online sexual risks among Taiwanese adolescents have provided important insights. For example, a study examining adolescents’ use of dating applications conceptualized such platform engagement as a high-risk online lifestyle and found that frequent use of dating apps, combined with unsafe online behaviors (e.g., sharing personal information, interacting with strangers), was significantly associated with heightened exposure to sexual solicitation and exploitation [28]. Related survey research has also shown that among Taiwanese adolescents, higher levels of online self-disclosure, longer time spent on instant messaging, and prior experiences of bullying significantly increase the likelihood of encountering unwanted online sexual solicitation (UOSS); by contrast, higher self-esteem appears to function as a protective factor [29]. These findings are consistent with the core propositions of Lifestyle–Exposure Theory, indicating that online routine activities, psychosocial vulnerabilities, and social-environmental factors jointly shape victimization risk [21,30].

Taken together, the empirical evidence from Taiwan suggests that Lifestyle–Exposure Theory can serve as an important analytical framework for understanding OGBV and intimate image offences; however, its application must be approached with caution to avoid shifting responsibility onto victims [31]. The observed associations among online routine activities, psychosocial distress, and victimization highlight the need for prevention strategies that focus on upstream interventions—namely, modifying opportunity structures for offending and strengthening institutional guardianship—rather than merely advising individuals to reduce their online engagement [25,27].

In the Taiwanese context, this includes strengthening school- and community-based digital literacy education [32], developing platform-level safeguards for image sharing [33], and enhancing institutional response capacities, such as establishing dedicated hotline services, constructing rapid takedown procedures, and training specialized police units capable of functioning as effective guardians in digital environments [7]. For the present study, these theoretical and empirical findings provide an important foundation for further examining how frontline professionals in Taiwan perceive and understand issues of risk, responsibility, and prevention when handling cases involving non-consensual intimate images.

### 2.2. Digital Technology and Gender-Based Violence

The rapid advancement of digital technology has significantly transformed both the forms in which gender-based violence occurs and the challenges associated with its handling, posing ongoing and concrete difficulties for frontline police and social work practice [1,31]. Technology-facilitated abuse (TFA) encompasses a range of behaviors, including the non-consensual dissemination of intimate images and online sexual extortion, which often occur through instantaneous and highly private digital communication channels. This places frontline professionals under considerable pressure to clarify facts and preserve evidence from the earliest stages of case handling [1,5].

In recent years, artificial intelligence (AI) has been increasingly employed in the generation and dissemination of images, particularly AI-generated child sexual abuse material (CSAM), which has further raised the technical threshold for image identification and case classification [34]. According to data from the Internet Watch Foundation (2024) [35], the number of confirmed CSAM reports that were actioned reached 291,273 webpages in 2024, representing a 6% increase compared to the previous year. These figures indicate that frontline operational units must contend with the dual pressures of rapidly increasing caseloads and highly compressed response timeframes [5,35].

At the operational level, OGBV frequently occurs within private instant messaging environments, such as Telegram, LINE, and WhatsApp [5]. Due to the widespread use of end-to-end encryption and closed-network architectures, police officers often must rely on victims to proactively provide screenshots, conversation records, or device content when collecting evidence, tracing perpetrators’ identities, and preserving digital evidence [22,36]. Social workers, similarly, must conduct crisis assessments, victim support, and referrals under conditions of considerable evidentiary uncertainty [37].

The decentralized and cross-border nature of these platforms also means that frontline professionals frequently encounter lengthy procedures, inconsistent responses, or jurisdictional limitations when requesting image takedowns or seeking platform assistance, further compromising the timeliness of victim protection [14,38]. These challenges underscore the need for clearer inter-agency protocols and more responsive platform cooperation mechanisms to support effective frontline practice.

In recent years, Taiwan has attempted to respond to crime patterns in digital contexts by amending the Criminal Code and related statutes, thereby providing clearer legal grounds for frontline practice [7,39]. However, practitioners continue to report that challenges persist across multiple dimensions, including case reporting, the application of criminal charges, inter-agency collaboration, and cross-jurisdictional requests. These challenges are often attributed to the fragmentation of legal provisions, insufficient operational guidelines, and uneven resource allocation [7,31].

For police officers, completing evidence collection and statutory procedures within limited timeframes while simultaneously ensuring victim protection constitutes a form of structural pressure in practice [40]. For social workers, assuming roles in emotional support, risk assessment, and accompaniment during the reporting process—often before legal proceedings have been clarified—significantly increases professional burden [37].

Overall, the practical challenges associated with technology-facilitated gender-based violence indicate that legislative amendments alone are insufficient to address frontline needs. Effective response strategies require the integration of cross-professional collaboration mechanisms, clear operational guidelines, responsive platform communication channels, and specialized training and support systems for both police and social work personnel. In this regard, Taiwan represents one of the earlier examples in Asia to adopt a multi-statute approach to regulating intimate image offences, which further underscores the value of examining how these reforms function in practice for international audiences. Accordingly, examining how institutional frameworks are understood, applied, and adapted in actual cases from the perspectives of frontline police and social work practice is of critical importance for evaluating Taiwan’s overall effectiveness in responding to non-consensual intimate images and OGBV.

### 2.3. Psychological Impacts and Victim Fear

Psychological trauma is a prevalent and serious consequence of online gender-based violence (OGBV), and systematic reviews of adolescents’ online sexual victimization and nonconsensual sharing of intimate images show elevated psychological distress, particularly symptoms of depression, anxiety, and suicidal ideation, alongside bullying and other negative social repercussions [2,41]. Compared with offline forms of violence, the enduring, hard-to-control circulation of digital content in cases of cyberbullying and image-based sexual abuse can prolong victims’ exposure to abuse and sustain or intensify feelings of fear, helplessness, and shame [42,43].

Theoretical and empirical studies further suggest that online hate speech and cyberbullying overlap substantially with OGBV in both etiology and psychological consequences, particularly in their long-term effects on emotional regulation and social functioning [44]. Importantly, victims’ anticipation of future revictimization—often conceptualised as fear of crime—can itself become a significant source of ongoing psychological distress, sometimes with more persistent effects on well-being than the initial victimization event [45]. Among adolescents, risks such as online sexual extortion, cyberstalking, and commercial exploitation have increasingly been recognised as major public health concerns, underscoring the need for interventions that integrate both preventive and therapeutic components [46].

International guidelines and local practice experience consistently indicate that secondary harm can be reduced, and victims’ psychological recovery and sense of safety can be better supported only through cross-professional collaboration and continuous, accessible support services [47,48]. From this perspective, psychological impacts and victim fear should be understood not as incidental consequences of individual cases, but as central public health concerns in evaluating institutional responses to OGBV and intimate image offences. In practice, victims frequently report persistent fear that intimate images may continue to circulate and that they may be re-identified or recognised by others [7,22]. These fears are closely associated with the enduring accessibility of digital material, the difficulty of tracing anonymous perpetrators, and the uncertainty of investigative and legal processes [14,36].

Practical experience also suggests that when institutional responses lack a trauma-informed approach—such as when excessive attention is paid to victims’ behaviour, victims are repeatedly asked to recount painful experiences, or case progress and risks are not clearly explained—feelings of shame, helplessness, and mistrust may be exacerbated, thereby reducing willingness to seek further help or cooperate with authorities [14,20]. By contrast, non-blaming communication, transparent procedural explanations, and timely linkage to psychological support and image takedown mechanisms are more likely to reduce fear and promote psychological stabilisation and restoration of safety [22,23,47]. Taken together, these considerations indicate that incorporating psychological harm and victim fear into core case-handling processes, and embedding trauma-informed principles through cross-sector collaboration, is essential to strengthening Taiwan’s institutional response to technology-facilitated gender-based violence.

## 3. Research Design and Methods

This study employed a qualitative research design to examine the effectiveness of Taiwan’s recent legal reforms and victim support measures addressing OGBV, with particular attention to offences involving intimate images. The research focused on the experiential knowledge and professional assessments of frontline practitioners who directly engage in case handling, law enforcement, prosecution, legal advocacy, and victim service delivery. The study design followed internationally recognized qualitative research standards to enhance transparency, methodological rigor, and analytical clarity [49,50,51].

### 3.1. Participants, Data Collection, and Ethical Considerations

A purposive sampling strategy was employed to recruit 20 professionals directly involved in the enforcement of intimate image offences and the provision of victim support services in Taiwan. The participant group included six social workers, six police officers, four lawyers, and four prosecutors, representing key stakeholders within both the criminal justice and victim assistance systems. Eligibility criteria required at least three years of relevant professional experience and direct involvement in cases concerning non-consensual dissemination of intimate images or other forms of technology-facilitated gender-based violence [52].

We employed purposive sampling to recruit information-rich cases, focusing on frontline professionals directly involved in the investigation, prosecution, and support of intimate image offences. This strategy was chosen to capture system-level implementation experiences during the early phase of legal reform.

Within these criteria, the researcher intentionally sought variation in professional role, institutional affiliation, years of service, gender, and case-handling experience to capture a wide range of perspectives across the organizational and legal hierarchy. Although the sample encompassed multiple institutional contexts, it was limited to selected regions in Taiwan and therefore does not represent all agencies within the national enforcement system.

Data were collected through semi-structured, in-depth interviews lasting approximately 60–90 min each, conducted either face-to-face or via secure online platforms depending on participant availability. An interview guide was developed to explore investigative procedures, evidentiary challenges, prosecutorial decision-making processes, interagency coordination mechanisms, legal advocacy strategies, and the accessibility and responsiveness of victim support services. With participants’ written informed consent, all interviews were audio-recorded and transcribed verbatim for subsequent qualitative content analysis.

This study was reviewed and approved by the Human Research Ethics Committee of National Chung Cheng University (Approval No. CCUREC114020701). Written informed consent was obtained from all participants prior to participation. Confidentiality was safeguarded through the use of identification codes and the removal of personally identifiable information from transcripts. All research data were securely stored and accessible only to the research team. In addition, data collection proceeded until thematic saturation was reached within each professional group; for example, no substantively new themes emerged after the interviews with the later social workers and police officers, indicating that the core patterns had been sufficiently captured across roles.

### 3.2. Conceptual Framework

**Figure 1:**
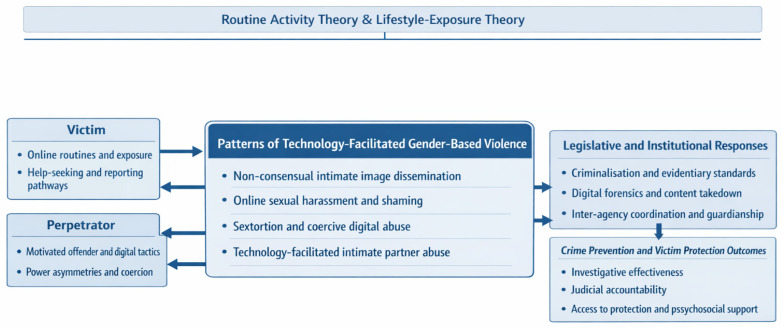
Conceptual Framework of Technology-Facilitated Gender-Based Violence. Note: Figure 1 presents the conceptual framework for technology-facilitated gender-based violence in Taiwan, showing how victim–perpetrator dynamics, patterns of digital and online violence, and legislative reforms interact, as well as their implications for criminal investigation, judicial processes, and protective measures. The arrows indicate the relationships among victim- and perpetrator-related factors, patterns of technology-facilitated gender-based violence, legislative and institutional responses, and crime prevention and victim protection outcomes. The blue-shaded boxes distinguish the major domains of the framework.

Building on Taiwan’s recent legal reforms addressing intimate image offences and broader forms of technology-facilitated gender-based violence (TFGBV), this study developed a theoretically informed conceptual framework to guide the organisation and analysis of qualitative data (Figure 1). The framework integrates Routine Activity Theory and Lifestyle-Exposure Theory and is designed to examine how interactional dynamics, patterns of digital violence, legislative and institutional responses, and prevention outcomes intersect within the criminal justice and victim support systems. Routine Activity Theory and Lifestyle-Exposure Theory informed both the conceptual framework and the coding strategy. We used RAT to identify opportunity structures and guardianship gaps within victim–perpetrator interaction dynamics, and LET to interpret how victims’ digital routines and exposure patterns shaped their vulnerability to image-based abuse.

The framework comprises four interrelated analytical dimensions: (1) victim–perpetrator interaction dynamics, including power asymmetries, digital coercion, and opportunity structures shaped by online routines; (2) patterns of technology-facilitated gender-based violence, such as non-consensual intimate image dissemination, online sexual harassment and shaming, sextortion and coercive digital abuse, and technology-facilitated intimate partner abuse; (3) legislative and institutional responses in Taiwan, including criminalisation standards, evidentiary and digital forensics practices, and inter-agency coordination mechanisms; and (4) crime prevention and victim protection outcomes, including investigative effectiveness, institutional guardianship, judicial accountability, and access to protective and psychosocial support services.

Figure 1 illustrates how these dimensions interact across macro (societal), meso (institutional), and micro (case-level) levels, and conceptualises the effectiveness of law enforcement and victim services as emerging from the alignment between legal frameworks, institutional practices, and lived case experiences. This framework functioned as an analytic scaffold that guided coding, category development, and cross-case comparison, and it is consistent with system-level qualitative approaches that foreground multi-stakeholder perspectives in research on technology-facilitated and online gender-based violence [22,53,54].

### 3.3. Data Analysis Method: Thematic Analysis

This study employed reflexive thematic analysis as the primary method for qualitative data analysis, following Braun and Clarke’s six-phase framework [54]. The analytic process was iterative, recursive, and closely grounded in the empirical data. The research team repeatedly reviewed the interview transcripts, conducted open coding to identify recurring patterns and meaningful units, and grouped related codes into candidate themes. Coding proceeded primarily inductively, while also being informed by the study’s conceptual framework. In particular, sensitising concepts derived from Routine Activity Theory (RAT) (e.g., motivated offenders, suitable targets, and absence of guardians) and Lifestyle-Exposure Theory (LET) (e.g., exposure to risky online environments) guided the development and refinement of thematic categories.

To enhance credibility and analytic rigour, the principal investigator and research assistants independently coded an initial subset of transcripts and resolved discrepancies through discussion, leading to refinement of the coding framework. Reflexive practices were incorporated throughout data collection and analysis to enhance transparency and reduce interpretive bias. Field notes were recorded after each interview to document initial impressions and emerging assumptions, and coding decisions and theme development were further reviewed through team discussions to consider alternative interpretations and reduce single-researcher dominance. An audit trail was maintained throughout the analytic process, and themes were reviewed against the full dataset to ensure that they were grounded in multiple participants’ accounts, including negative cases and divergent perspectives. Data collection and analysis proceeded concurrently until no substantively new themes emerged, indicating theoretical saturation. These procedures enhanced the transparency, credibility, confirmability, and analytic rigour of the findings despite the modest sample size.

### 3.4. Research Tools

Data were collected using an in-depth semi-structured interview guide designed to explore frontline professionals’ experiences with the enforcement of intimate image offence legislation and the provision of victim support services in Taiwan. The guide was developed to elicit detailed accounts of legal implementation, institutional practice, and victim protection in cases involving online gender-based violence (OGBV).

Prior to formal data collection, the interview guide was pilot-tested with two professionals from relevant fields. Minor revisions were made to improve the clarity and sequencing of the questions.

The final interview guide covered seven main domains: (1) practical challenges in handling intimate image offence cases; (2) operational barriers related to legal fragmentation and statutory implementation; (3) digital evidence collection, content removal mechanisms, and cross-platform enforcement constraints; (4) experiences with victim protection measures, including protective orders and compensation schemes; (5) risks of secondary victimization within investigative and judicial procedures; (6) inter-agency coordination practices and structural gaps; and (7) recommendations for strengthening victim-centred, trauma-informed institutional responses.

The guide combined open-ended questions with focused probes to encourage detailed professional reflections while maintaining alignment with the study’s conceptual framework.

### 3.5. Strategies for Ensuring Trustworthiness

Given the sensitive and legally complex nature of technology-facilitated sexual violence, several strategies were adopted to enhance the credibility, dependability, and confirmability of the findings. First, data and role-based triangulation were achieved by including social workers, police officers, prosecutors, and lawyers, with variation in gender, years of service, and case-handling experience, allowing cross-role comparison of legal interpretation, enforcement practices, and victim protection mechanisms.

Second, the principal investigator and research assistants engaged in regular peer debriefing and collaborative coding, discussing contested interpretations—particularly those related to institutional accountability, legal fragmentation, and secondary victimisation—to avoid single-perspective dominance. Third, reflexive notes and team discussions were used to monitor how normative commitments to victim protection and legal reform might shape analysis, helping to keep interpretations grounded in the data rather than advocacy positions.

Finally, attention was given to deviant cases and contradictory perspectives, especially where participants questioned the effectiveness of reforms or described dissatisfaction with image removal, compensation, or protective orders. These procedures were implemented to strengthen the transparency and balance of the qualitative analysis in a field characterised by legal complexity, technological volatility, and trauma-sensitive practice.

## 4. Results

### 4.1. Research Results and Analytical Overview

Prior to presenting the detailed thematic findings, we provide a brief analytical overview of the interview corpus based on our coding and reflexive engagement with the data. This overview highlights how frontline practitioners recurrently described victims, online gender-based violence, and legal–institutional responses.

**Figure 2:**
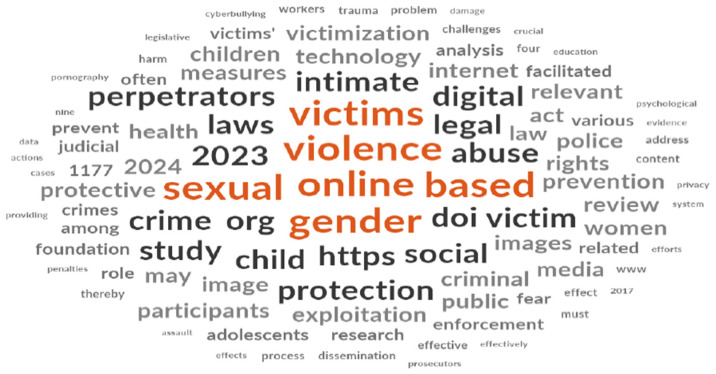
Word Cloud of Respondents’ Terms.

The salience of these clusters is consistent with the study’s conceptual framework and helps situate the subsequent qualitative findings. The core analysis and interpretations are derived from the reflexive thematic analysis presented in the following sections and are visually synthesized in the conceptual framework (Figure 1) and the thematic map (Figure 2).

### 4.2. Structural Challenges in Taiwan’s Response to Digital Sexual Violence

#### 4.2.1. Superordinate Themes of Enforcement and Victim Service Gaps

Through reflexive thematic analysis, the interview data were organised into three superordinate themes that capture the structural tensions between legislative intent, institutional implementation, and victim-centred protection outcomes. These themes reflect how enforcement and victim protection challenges operate across macro-, meso-, and micro-level contexts in cases of digital sexual violence.

Participants consistently indicated that, although recent legal reforms have strengthened formal protections, substantial gaps remain in public awareness, institutional coordination, and victim-centred procedural responsiveness. Rather than appearing as isolated problems, these challenges were described as interconnected and mutually reinforcing, collectively shaping the overall effectiveness of legal enforcement and victim services.

As shown in Table 2, the three superordinate themes were: (1) public awareness deficits and cultural normalization; (2) legal fragmentation and implementation barriers; and (3) gaps in victim-centred protection and remedies. Together, these themes illustrate the multi-level barriers that continue to affect the implementation of intimate image offence legislation and the delivery of victim support in Taiwan.

#### 4.2.2. Theme 1: Public Awareness Deficits and Cultural Normalization

The first superordinate theme highlights the persistent gap between legislative reform and public awareness. Although Taiwan has enacted significant amendments to criminalize intimate image offences, participants reported that public knowledge of these legal changes remains limited. One prosecutor noted that “not everyone is aware of the new legislative amendments, which is a significant problem. The government lacks effective means of promoting amendments and implementing new laws” (Prosecutor-002).

Participants further emphasized that deeply rooted cultural norms surrounding digital image sharing contribute to the normalization of harmful behaviors. A social worker explained that societal attitudes often shift responsibility toward victims rather than perpetrators, noting that “in the past, society criticized victims instead of addressing perpetrators or institutions” (Social Worker-005).

Additionally, normalization of digital practices and technological familiarity has reduced perceived risks associated with image sharing and dissemination. As another social worker observed, “if individuals consider secretly capturing intimate images as normal, this mindset is detrimental to the societal environment” (Social Worker-006).

These findings suggest that legal reform alone cannot effectively prevent digital sexual violence without corresponding changes in public awareness, digital literacy, and cultural attitudes. This theme corresponds to the macro-level dimension of the conceptual framework, emphasizing the influence of societal context on enforcement effectiveness.

#### 4.2.3. Theme 2: Legal Fragmentation and Implementation Barriers

The second superordinate theme reflects structural challenges embedded within Taiwan’s legal and institutional framework. Participants consistently reported that relevant legal provisions are dispersed across multiple statutes, creating interpretive complexity and procedural inefficiencies.

A prosecutor explained that “navigating multiple interconnected laws is cumbersome and inefficient” (Prosecutor-004), while a lawyer noted that “we cannot expect everyone involved in a case to fully understand all interconnected laws” (Lawyer-008). Similarly, a social worker observed that “legal provisions are too dispersed. Navigating them is challenging even for professionals” (Social Worker-017).

In addition to legislative fragmentation, participants emphasized challenges related to digital evidence collection and preservation. Digital content can be easily altered, deleted, or redistributed, complicating investigative procedures and evidentiary verification.

Institutional coordination challenges further weaken enforcement capacity. As one police officer explained, “you might overlook relevant laws, risking failure to protect victims” (Police Officer-014). This reflects systemic limitations in inter-agency communication and procedural clarity.

### 4.3. Victim-Centered Protection Gaps and Procedural Challenges

#### 4.3.1. Limitations in Victim Protection Mechanisms

The third superordinate theme centers on victim experiences within the legal and institutional response system. Participants consistently emphasized that victims prioritize rapid content removal and prevention of further harm.

A lawyer explained that “victims’ primary concern is preventing further dissemination of intimate images” (Lawyer-016), underscoring the urgency of intervention in digital contexts where harm can rapidly escalate.

Similarly, a prosecutor identified victims’ primary needs as “addressing harm, uncovering truth, and preventing further damage” (Prosecutor-010), highlighting the multidimensional nature of victim recovery and protection.

However, participants reported that existing legal procedures often fail to provide timely protection. Procedural delays, evidentiary requirements, and jurisdictional constraints limit the speed and effectiveness of institutional responses.

#### 4.3.2. Secondary Victimization and Institutional Trust

Participants also emphasized the risk of secondary victimization within the justice process. A social worker explained that “victims may hesitate to seek assistance due to retraumatization within the judicial system” (Social Worker-019), indicating that legal procedures themselves can exacerbate psychological harm.

Similarly, participants expressed concerns regarding the adequacy of compensation mechanisms and legal penalties. A police officer noted that “legal penalties do not adequately reflect victims’ suffering” (Police Officer-014), suggesting that victims may perceive justice outcomes as insufficient.

#### 4.3.3. Integration Across Structural Levels

Taken together, the findings indicate that enforcement effectiveness in digital sexual violence cases is shaped by the interaction of macro-, meso-, and micro-level factors. At the macro level, societal awareness, cultural norms, and digital literacy influence how harm is recognised, how victims are perceived, and whether reporting is encouraged or discouraged. At the meso level, institutional design, legislative fragmentation, and enforcement capacity affect the consistency and effectiveness of legal implementation, including digital evidence collection, inter-agency coordination, and platform engagement. At the micro level, victim-centred procedural responsiveness shapes protection outcomes, particularly in relation to timely content removal, prevention of secondary victimization, and access to supportive remedies. Considered together, these levels demonstrate that the effectiveness of enforcement and victim protection depends not only on legal reform itself, but also on the alignment between broader social norms, institutional capacity, and the lived procedural experiences of victims.

## 5. Discussion

### 5.1. Structural Gaps Between Legislative Reform and Enforcement Effectiveness

This study shows that, although recent reforms in Taiwan have expanded criminal liability and formal protections for victims of intimate image offences, legal change alone has not translated into robust enforcement. Enforcement effectiveness is constrained by fragmented provisions across multiple statutes, uneven operational guidance, and limited institutional capacity, echoing international research on the difficulty of adapting traditional criminal law to fast-evolving digital harms. These findings are consistent with institutional perspectives that emphasise the need for organisational adaptation, clear procedures, and capacity-building—rather than statutory amendments alone—to produce meaningful frontline change.

These findings echo international studies on image-based abuse that document similar tensions between progressive legal reform and uneven implementation; however, they extend this work by providing system-level insights from a civil-law East Asian context.

Taken together, these accounts suggest a mechanism whereby fragmented legislation and limited coordination generate procedural uncertainty, which in turn discourages proactive enforcement and undermines victim trust.

### 5.2. Societal Awareness, Cultural Norms, and Enforcement Outcomes

The results highlight how low public awareness and persistent victim-blaming norms weaken prevention and reporting despite formal criminalisation. This pattern mirrors global evidence that TFSV is embedded in gendered power relations and social perceptions that minimise harm and discourage help-seeking. From a routine activity perspective, digital environments heighten target visibility and reduce guardianship, while limited digital literacy and misconceptions about consent blunt the deterrent effect of law. These dynamics underscore the need to complement legal reform with public education, digital citizenship initiatives, and prevention strategies that explicitly challenge victim-blaming and support victim-centred responses.

Our results align with prior research on technology-facilitated sexual violence, showing that victim-blaming norms and low digital literacy undermine reporting and help-seeking, while also highlighting how these mechanisms operate within Taiwan’s emerging digital governance framework.

### 5.3. Institutional and Technological Barriers to Effective Enforcement

At the meso level, participants reported difficulties in collecting and preserving digital evidence, securing platform cooperation, and coordinating across jurisdictions. These challenges reflect a structural mismatch between rapidly changing digital technologies and slower institutional adaptation, and they resonate with international studies that identify evidentiary complexity, limited cross-border cooperation, and uneven platform responsiveness as core obstacles in tackling online sexual violence. Fragmented responsibilities among agencies further contribute to delays and procedural uncertainty. The findings point to the need for integrated institutional frameworks, specialised digital investigation units, and clearer coordination mechanisms to strengthen enforcement capacity.

### 5.4. Victim-Centred Protection and Risks of Secondary Victimisation

At the micro level, the findings reveal gaps between legal protections on paper and victims’ experiences in practice. Participants noted that victims prioritise rapid content removal and prevention of further circulation, yet existing procedures do not consistently provide timely or effective intervention. This is consistent with victimological and public health research emphasising procedural justice, trauma-informed practice, and victim-centred responses in TFSV cases. Delayed takedown, repeated retelling of events, and complex legal procedures can intensify psychological distress and erode trust in institutions, reinforcing international evidence on secondary victimisation in digital sexual violence proceedings. These results underscore the need for trauma-informed institutional practices, specialised training, streamlined procedures, and accessible psychosocial and legal support throughout the case trajectory.

The interviews indicate that low public awareness and persistent stigma operate as key mechanisms that suppress reporting and limit the visibility of intimate image offences within the criminal justice system.

### 5.5. Multi-Level Structural Model of Digital Sexual Violence Response

Taken together, the findings support a multi-level structural model in which enforcement effectiveness emerges from the interaction of macro-level societal factors, meso-level institutional structures, and micro-level victim experiences. At the macro level, public awareness, gender norms, and digital literacy influence both reporting behaviour and recognition of harm; at the meso level, legal coherence, institutional coordination, and technological capacity shape investigative and prosecutorial performance; at the micro level, the responsiveness and sensitivity of procedures affect victim recovery, institutional trust, and perceived justice outcomes. This model is consistent with ecological and systems-based approaches to violence prevention and public health, which emphasise that legal rules alone cannot explain outcomes without attention to implementation processes and lived experiences.

### 5.6. Policy Implications

Building on this multi-level analysis, several policy directions emerge. Legislative integration and clarification are needed to reduce fragmentation, improve consistency in interpretation, and provide clearer guidance to frontline practitioners. Investment in specialised digital investigation units, digital forensics tools, and standardised evidence-preservation protocols is essential to address technological and cross-jurisdictional challenges. Improved inter-agency coordination mechanisms—such as permanent cross-ministerial task forces or coordination hubs for image takedown and platform engagement—could reduce delays and enhance institutional coherence. Trauma-informed, victim-centred protection systems should be institutionalised through regular training for police, prosecutors, judges, and social workers, simplified procedures, and expanded access to psychological, legal, and social support. Finally, sustained public awareness campaigns and digital citizenship education are critical to prevent TFSV, counter victim-blaming, and support help-seeking, aligning legal reforms with broader public health strategies.

### 5.7. Contribution to the Literature

This study contributes to the growing literature on TFSV by providing an empirically grounded, system-level analysis of enforcement and victim protection in a civil-law, East Asian context. By integrating qualitative interview data with a multi-level conceptual framework informed by Routine Activity Theory and Lifestyle-Exposure Theory, it demonstrates how legal reform, institutional capacity, and sociocultural context interact to shape enforcement effectiveness. These patterns are consistent with Routine Activity Theory, which suggests that increased online visibility combined with weak institutional guardianship facilitates opportunities for digital sexual offending.

The findings extend existing research by highlighting the mechanisms through which legislative fragmentation, institutional constraints, and cultural norms mediate the relationship between “law on the books” and “law in action,” and by underscoring the centrality of implementation processes and victim-centred protection mechanisms for translating legal reforms into meaningful public health and justice outcomes.

Framing these findings within a public health paradigm highlights that strengthening criminal justice responses to image-based abuse is also a strategy for reducing population-level mental health burdens and improving access to trauma-informed care.

## 6. Conclusions

This study examined Taiwan’s institutional responses to intimate image offences through qualitative interviews with frontline professionals. Using a multi-level analytical framework, the findings reveal that enforcement effectiveness and victim protection are shaped not only by legal reform but also by the interaction of societal norms, institutional structures, and victim-centered practices. While Taiwan has advanced in criminalizing non-consensual image dissemination, persistent barriers—including limited public awareness, fragmented legislation, and inadequate inter-agency coordination—continue to weaken prevention and protection efforts.

From a public health perspective, technology-facilitated sexual violence (TFSV) should be recognized as a population-level health concern rather than a purely legal issue. Consistent with ecological and multi-level models of violence prevention, the study highlights that risk and harm are influenced by social attitudes, institutional capacities, and individual psychological responses. Victims commonly experience anxiety, fear, and trauma, underscoring the broader mental health impact associated with TFSV and the need for integrated care pathways.

Addressing these challenges requires coordinated strategies that extend beyond statutory reform. Primary prevention should focus on public education and digital literacy to foster consent and bystander engagement; secondary prevention should strengthen early detection, evidence preservation, and rapid takedown mechanisms; and tertiary prevention must ensure trauma-informed, victim-centered support across legal, health, and social sectors. Developing a cohesive public health framework that bridges justice, welfare, and digital governance systems is essential to enhance institutional responsiveness and promote population-level mental well-being.

## 7. Research Limitations

This study was conducted between August 2025 and January 2026, relatively soon after the 2023 reforms came into effect. As a result, the findings primarily capture an early implementation stage in which institutional practices and police protocols were still evolving through “learning by doing.”

In addition, the study relies on a small, information-rich qualitative sample of 20 interviews drawn from specific regions and mainly from criminal justice and formal service institutions. This design limits national generalisability and may introduce institutional bias, particularly given the absence of survivors’ direct perspectives and more systematic input from grassroots NGOs. Consequently, the findings and policy implications should be interpreted as exploratory and context-bound and require further corroboration through larger, mixed-methods and survivor-centred research.

The qualitative sample is not statistically representative and is limited to professional stakeholders from specific regions, which restricts national generalisability and may introduce institutional bias. Furthermore, the absence of survivors’ voices means that the study can only offer an indirect account of victim experiences, and future survivor-centred research is needed to evaluate service effectiveness more fully.

## 8. Future Research

Future research should prioritise survivor-centred designs to complement the system-level professional perspectives reported in this study. In particular, in-depth qualitative or longitudinal studies with survivors of intimate image abuse are needed to examine how they experience reporting processes, access to support services, and justice outcomes over time. Collaborations with specialist NGOs and community-based organisations could facilitate ethical recruitment, enhance safeguards against retraumatisation, and ensure that research protocols are grounded in trauma-informed practice. In addition, mixed-methods designs that integrate survivor narratives with institutional and administrative data would allow a more comprehensive assessment of service accessibility, institutional responsiveness, and the effectiveness of recent legal reforms from the victims’ standpoint.

## Figures and Tables

**Table 1:** The Four Acts on Sexual Violence Prevention in Taiwan and key 2023 amendments on digital sexual and gender-based violence.

Act	Core Regulatory Focus	Key 2023 Amendments (Digital Sexual and Gender-Based Violence)
Criminal Code	Substantive sexual offences and penalties.	Introduced an offence for non-consensual dissemination of sexual images (6 months–5 years’ imprisonment), a specific provision on deepfake sexual imagery (up to 7 years), and increased penalties where images are produced or disseminated for profit.
Sexual Assault Crime Prevention Act	Reporting, investigation, and victim protection in sexual assault cases.	Established an immediate takedown mechanism for illegal sexual images, empowered prosecutors and courts to order URL blocking, and strengthened victim support for searching and removing non-consensual sexual images online.
Child and Youth Sexual Exploitation Prevention Act	Sexual exploitation of children and youth, including pornography and related conduct.	Clarified that production, distribution, or possession of sexual images of minors is unlawful even if “voluntarily” created, and increased penalties for purchasing or possessing child sexual images, including possession without legitimate purpose.
Crime Victim Rights Protection Act	Rights, compensation, and support services for crime victims.	Enhanced privacy protections to restrict dissemination of sexual images and expanded access to psychological counselling, legal assistance, and related support for victims of digital sexual violence.

Note. This table is compiled by the authors based on official descriptions of the 2023 amendments to the four statutes.

**Table 2:** Superordinate themes and subthemes related to enforcement and victim protection gaps in digital sexual violence cases.

Superordinate Theme	Subtheme
Public Awareness Deficits and Cultural Normalization	Limited dissemination of new laws Persistent victim-blaming attitudes
Legal Fragmentation and Implementation Barriers	Dispersion across multiple statutes Platform and digital investigation barriers Evidentiary and procedural complexity
Gaps in Victim-Centred Protection and Remedies	Delays in content removal Risk of secondary victimization Dissatisfaction with compensation

Note. The table presents the thematic structure derived from reflexive thematic analysis. Illustrative quotations for each subtheme are presented in the corresponding Results Section 4.

## Data Availability

The data presented in this study are available upon request from the corresponding author. The data are not publicly available due to privacy and ethical considerations.

## Abbreviations

OGBV	Online Gender-Based Violence
TFA	Technology-Facilitated Abuse
TFSV	Technology-Facilitated Sexual Violence
NCDSI	Non-Consensual Dissemination of Sexual Images
RAT	Routine Activity Theory
CSAM	Child Sexual Abuse Material
